# Pioneer transcription factors are associated with the modulation of DNA methylation patterns across cancers

**DOI:** 10.1186/s13072-022-00444-9

**Published:** 2022-04-19

**Authors:** Roza Berhanu Lemma, Thomas Fleischer, Emily Martinsen, Marit Ledsaak, Vessela Kristensen, Ragnhild Eskeland, Odd Stokke Gabrielsen, Anthony Mathelier

**Affiliations:** 1 Centre for Molecular Medicine Norway (NCMM), Nordic EMBL Partnership, University of Oslo, Oslo, Norway; 2 Department of Cancer Genetics, Institute for Cancer Research, Oslo University Hospital, Oslo, Norway; 3 Institute of Basic Medical Sciences, Department of Molecular Medicine, and Centre for Cancer Cell Reprogramming, Institute of Clinical Medicine, Faculty of Medicine, University of Oslo, Oslo, Norway; 4 Department of Medical Genetics, Oslo University Hospital, Oslo, Norway; 5 Institute of Clinical Medicine, Faculty of Medicine, University of Oslo, Oslo, Norway; 6 Department of Biosciences, University of Oslo, Oslo, Norway

## Abstract

Methylation of cytosines on DNA is a prominent modification associated with gene expression regulation. Aberrant DNA methylation patterns have recurrently been linked to dysregulation of the regulatory program in cancer cells. To shed light on the underlying molecular mechanism driving this process, we hypothesised that aberrant methylation patterns could be controlled by the binding of specific transcription factors (TFs) across cancer types. By combining DNA methylation arrays and gene expression data with TF binding sites (TFBSs), we explored the interplay between TF binding and DNA methylation in 19 cancer types. We performed emQTL (expression–methylation quantitative trait loci) analyses independently in each cancer type and identified 13 TFs whose expression levels are correlated with local DNA methylation patterns around their binding sites in at least 2 cancer types. The 13 TFs are mainly associated with local demethylation and are enriched for pioneer function, suggesting a specific role for these TFs in modulating chromatin structure and transcription in cancer patients. Furthermore, we confirmed that de novo methylation is precluded across cancers at CpGs lying in genomic regions enriched for TF binding signatures associated with SP1, CTCF, NRF1, GABPA, KLF9, and/or YY1. The modulation of DNA methylation associated with TF binding was observed at cis-regulatory regions controlling immune- and cancer-associated pathways, corroborating that the emQTL signals were derived from both cancer and tumor-infiltrating cells. As a case example, we experimentally confirmed that FOXA1 knock-down is associated with higher methylation in regions bound by FOXA1 in breast cancer MCF-7 cells. Finally, we reported physical interactions between FOXA1 with TET1 and TET2 both in an in vitro setup and in vivo at physiological levels in MCF-7 cells, adding further support for FOXA1 attracting TET1 and TET2 to induce local demethylation in cancer cells.

**Supplementary Information:**

The online version contains supplementary material available at 10.1186/s13072-022-00444-9.

## Introduction

Chromatin and DNA modifications act as molecular stamps associated with active and inactive regulatory status of corresponding genomic regions, which are crucial for proper homeostasis and development [1, 2]. Among the various possible DNA modifications [3], the addition of a methyl group to the 5th carbon of cytosine leads to the 5-methylcytosine (5mC) mark. The 5 mC mark (hereafter referred to as DNA methylation) is usually associated with the transcriptional silencing of cis-regulatory elements, such as promoters or enhancers [4, 5]. As aberrant DNA methylation patterns are linked to various diseases, such as cancers [6–8], it is critical to understand the underlying molecular mechanisms driving this process.

Covalent DNA methylation at cytosines (mainly in the CpG context) is acquired by the addition of 5-methylcytosine catalysed by the DNA methyltransferase (DNMT) enzymes. DNA demethylation is carried out by the Ten–Eleven Translocation (TET) proteins in successive hydroxylation reactions resulting in 5mC derivatives, which are removed by thymine DNA glycosylase through the base excision repair pathway (reviewed in [9]). As DNMTs and TETs bind DNA in a limited sequence-specific manner, their recruitment to specific genomic regions has been reported to be driven by interactions with transcription factors [10–13].

Transcription factors (TFs) are proteins that recognize and bind cis-regulatory regions (promoters and enhancers) at their TF binding sites (TFBSs) through sequence-specific TF–DNA interactions to regulate transcription [14]. Through their binding at cis-regulatory regions, most TFs recruit co-factors to activate or repress the transcription of target genes [14, 15]. While most of the TFs engage with open chromatin regions at their TFBSs, a specific class of TFs, the pioneer TFs, have the ability to engage with nucleosome-bound chromatin independent of other factors. Pioneer TFs are believed to be the first factors to engage with target chromatin regions and associate with compact chromatin to facilitate the binding of other additional factors and local epigenetic modifications [16–18]. For instance, changes across myeloid cell fate transitions are marked with the priming of inaccessible enhancers by pioneer TFs, which leads to locally increased chromatin accessibility and DNA methylation loss [19].

Several TFs have been reported to physically interact with DNMTs and/or TETs and are, therefore, likely to recruit these enzymes to specific genomic regions. The leukemogenic PML–RAR fusion protein has been shown to recruit DNMTs, while RUNX1 recruits the DNA demethylation machinery [20–22]. Using co-immunoprecipitation in HEK293T cells and endogenous IP in LNCaP cells, FOXA1 was found to physically interact with TET1 and promote the co-occupancy of TET1 in FOXA1 occupied regions [23].

To investigate the association between TF binding and DNA demethylation at large scale, Suzuki et al*.* developed a screening system combined with TF binding motif enrichment at differentially methylated regions after ectopic expression of selected TFs. This strategy identified a set of developmental (cell fate determining) TFs that were associated with binding site-directed DNA demethylation [24]. Another high-throughput screening strategy investigated the interplay between TF binding and DNA methylation for hundreds of TFs [25]. The strategy relies on the integration of a sequence backbone with known methylation status but containing diverse TF binding motifs followed by bisulfite sequencing of PCR amplicons. The study revealed pioneer TFs that can induce local DNA demethylation and pioneer TFs whose binding have a protective effect against de novo DNA methylation [25]. Using a computational approach, the ELMER (Enhancer Linking by Methylation/Expression Relationships) tool allowed for the large-scale identification of transcriptional enhancers and their target genes based on DNA methylation data (at enhancers) and gene expression [26]. Motif enrichment analysis at the enhancers predicted pan-cancer by ELMER inferred TFs that could act as upstream regulators of DNA methylation patterns at these enhancers [26]. Similarly, the TENET framework identified cancer-specific hypo- and hyper-methylated CpGs in putative enhancers before linking them with candidate upstream regulators through methylation–expression correlation [27]. Using this strategy, TENET predicted > 1200 TFs potentially regulating enhancer networks in breast, prostate, and kidney cancers [27]. Despite continuous efforts to unravel the molecular mechanism by which DNA methylation is regulated, the current understanding of how DNA methylation is regulated and its interplay with TF binding in cancer patients is limited [19, 22, 24]. We hypothesised that a pan-cancer and genome-wide investigation of the interplay between TF binding and resulting local DNA methylation patterns in cancer genomes could reveal key regulatory processes that are critical for an improved molecular understanding of cancers.

In this study, we designed a computational approach to identify CpGs with DNA methylation level correlated with the expression level of 231 TFs. We further assessed the enrichment of these CpGs around TFBSs for the corresponding TFs. This TF binding-centric expression–methylation quantitative trait loci (emQTL) methodology was applied to 19 cancer types from The Cancer Genome Atlas (TCGA) to predict TFs associated with DNA methylation patterns (emTFs, expression–methylation TFs). The analyses revealed 13 emTFs (33 TF-cancer type pairs) for which an enrichment for correlated CpGs around their TFBSs was observed in at least 2 cancer types, providing evidence for their potential role in DNA methylation patterns in cancer patients. The pioneer function of these 13 emTFs, which we found predominantly associated with DNA demethylation, has been demonstrated by previous studies. Furthermore, we confirmed the presence of TF binding signatures that are discriminative between regulatory regions associated with varying DNA methylation across patients and regions, where de novo DNA methylation is precluded. From the list of 13 emTFs, we experimentally investigated the role of FOXA1 in DNA demethylation in breast cancer. We observed that FOXA1 knockdown led to an increase of DNA methylation at some regions bound by FOXA1 in MCF-7 cells. We further reported physical interactions between FOXA1 and both TET1 and TET2 at physiological levels in MCF-7 cells as well as using in vitro GST-pulldown assays.

## Results

### Prediction of transcription factors associated with DNA methylation patterns around their binding sites across cancer types

We aimed to unravel the interplay between TF binding to the DNA and local DNA (de-)methylation. We hypothesised that the binding of specific TFs to their TFBSs would be correlated with local DNA (de-)methylation if these factors were associated with DNA modifications. By combining DNA methylation (from Illumina 450 K arrays) and gene expression data from 19 cancer type cohorts from TCGA [28] (Additional file 4: Table S1) with high-quality direct TF–DNA interactions (i.e. TFBSs) from the UniBind database [29], we assessed the correlation between DNA methylation and TF binding using TF expression as a surrogate for TF binding potential at their TFBSs. Altogether, we evaluated the expression of 231 TFs with DNA methylation at CpGs in cancer cohorts of 59 to 703 patients (Additional file 4: Table S1). Specifically, we performed expression–methylation quantitative trait loci (emQTL) analyses by computing Spearman correlation coefficients between the expression of the 231 TFs and methylation level at 376,997 CpGs located close to TFBSs in each cancer type independently (see "Materials and methods" section for details and Additional file 4: Table S2 for the number of CpGs close to TFBSs for each TF). This emQTL computation followed our previously published methodology associating CpGs with gene expression [8] but was restricted to TFs and CpGs surrounding their binding sites. Note that for each TF, we considered all 376,997 CpGs in the emQTL analysis.

**Fig. 1:**
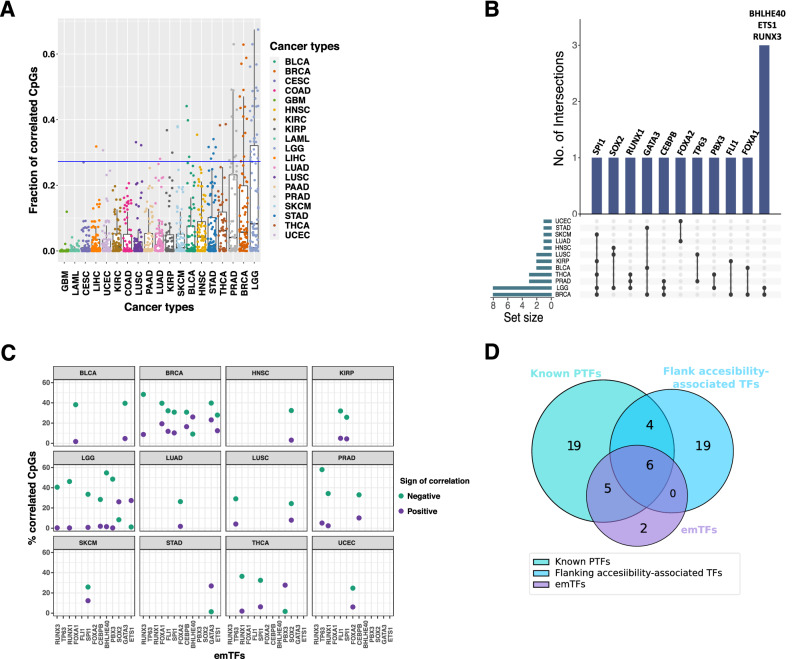
Identification and analysis of emTFs. **A** Box plot depicting the fraction of CpGs close to TFBSs for each TF (each point corresponds to a TF in a given cohort (columns)) with DNA methylation level correlated with the TF expression. The horizontal blue line represents the 95th percentile of the distribution of all fractions (see Additional file 3: Fig. S1C for the distribution). **B** Upset plot representing the emTF predictions across the cancer types. Each row represents a cancer type with points providing information about the intersection of the TFs predicted in the different cancer types. The bars at the top indicate the number of intersecting TFs (annotated above each bar) in each combination of cancer types (indicated by the points). The set size below the horizontal bars depicts the number of TFs predicted in each cohort. **C** For each emTF (columns), the plot provides the percentage of positively (purple dots) and negatively (green dots) correlated CpGs (emCpGs; *y*-axis) close to their TFBSs predicted in each cancer type (one per facet). See Additional file 4: Table S3 for the detailed numbers. **D** Venn diagram of the intersection between the predicted emTFs (*n* = 13), pioneer TFs from the literature (known PTFs; *n* = 34), and flanking accessibility-associated TFs from [43] (*n* = 29)

For each TF we examined the proportion of the CpGs close to its TFBS that were in emQTL with the TF itself; the percentages varied significantly between TFs and across cancer types (Fig. 1A). In some cancer types, several TFs were associated with high percentages of correlated CpGs (e.g. in breast cancer, BRCA, and brain lower grade glioma, LGG), while small proportions of CpGs were observed for all TFs in other cancer types (e.g. in glioblastoma multiforme, GBM, and acute myeloid leukaemia, LAML). We examined whether this variability could be explained by the lack of statistical power in the emQTL analyses for the cohorts with a lower number of samples. Indeed, we observed a significant correlation between the number of samples in a cohort and the median number of correlated CpG percentages (Additional file 3: Fig. S1B). We speculate that the identification of TFs that could be associated with local DNA methylation patterns around their TFBSs is precluded in cohorts with smaller sample sizes.

To focus on the TFs for which the binding is most likely to have a local effect on DNA methylation, we considered the TFs associated with the highest percentages of correlated CpGs that were especially enriched close to their TFBSs. Specifically, we extracted the top 5% of CpG percentages from the distribution obtained for all TF-cancer pairs (Additional file 3: Fig. S1C). In addition, we filtered out TFs that did not show a specific enrichment of CpGs in emQTL close to their TFBSs. The filtering was achieved by assessing the enrichment for correlated CpGs around the TF’s TFBSs using Mann–Whitney *U* tests; we retained TFs with *p* values < 0.01 ("Materials and methods" section; Additional file 4: Table S3). This strategy revealed 37 TFs in 12 cancer types (Additional file 3: Fig. S1D). We observed consistent association with local DNA methylation patterns in at least 2 cancer types for 13 TFs (Fig. 1B). Even though the 13 TFs were associated with an enrichment of correlated CpGs close to their binding sites, the corresponding CpGs identified in each cancer type vary (Additional file 3: Figs. S2, S3). Hereafter, we refer to these 13 TFs as emTFs (expression–methylation TFs) and to the correlated CpGs close to their TFBSs as emCpGs (expression–methylation CpGs).

Cytosines represented in the Illumina 450 K methylation array are not distributed evenly throughout the genome but mainly localised in proximal promoters and gene bodies [30]. Similarly, the TFBSs from UniBind that were considered in this study are also predominantly found at proximal promoters [31]. We assessed the genomic distribution of emCpGs and compared it to the complete set of 376,997 CpGs considered (and located close to TFBSs). Across cancer types, we observed a smaller proportion of emCpGs at proximal promoters than observed with the complete set of CpGs, while emCpGs were more frequently found at intronic and intergenic regions (Additional file 3: Fig. S4). This observation suggests that emCpGs are more predominantly detected at distal regulatory elements than promoter regions.

### emTFs are mainly associated with demethylation and are enriched for pioneer function

We sought to provide molecular mechanistic insights underlying the interplay between emTF binding and local DNA methylation modulation around their TFBSs. We first investigated the nature of the correlations (positive versus negative correlations) between emTFs’ expression and DNA methylation at emCpGs. Across cancer types, the expression of the emTFs was mainly negatively correlated with the level of methylation of the associated emCpGs (Fig. 1C; Additional file 3: Fig. S5). The proportion of negatively correlated emCpGs ranged from ~ 1 to ~ 58% per TF-cohort (mean = 30.7%; median = 32.1%), while the proportion of positively correlated emCpGs ranged from ~ 0.3 to ~ 28% (mean = 9.6%; median = 6%). These results indicate that, in most cases, higher emTF expression is associated with lower CpG methylation around their TFBSs, suggesting local DNA demethylation through TF binding.

Higher level of DNA methylation is usually associated with silenced and inaccessible cis-regulatory regions [4, 32]. We speculated that the emTFs would engage with these regions of methylated and closed chromatin to trigger demethylation and chromatin accessibility. As pioneer TFs have the capacity to engage with closed chromatin, we examined if the 13 identified emTFs were enriched for such pioneer function. We collected a list of pioneer TFs by reviewing the literature (Additional file 4: Table S4) [33–42] and found that the emTFs were enriched in the list of pioneer TFs (11 out of the 13 emTFs; Fisher test *p* value < 9.4e^−31^; Fig. 1D).

Next, we aimed to provide complementary evidence for emTFs to engage with closed chromatin and reshape the chromatin landscape in cancer patients. A recent study reported the chromatin accessibility landscape of human cancers using ATAC-seq [43]. This work predicted 55 TFs (29 of which were among the 231 TFs investigated in this study) for which the binding is associated with increased chromatin accessibility in the regions flanking their TFBSs, providing evidence for their pioneer function [43, 44]. We found that the emTFs were enriched in the list of flanking accessibility-associated TFs reported from cancer samples in [43] (6 out of the 13 emTFs: CEBPB, GATA3, FOXA1, RUNX1, RUNX3, and TP63; Fisher test *p* value < 3.9e^−15^; Fig. 1D; Additional file 4: Table S5).

Furthermore, the ATAC-seq study observed that the increased chromatin accessibility was accompanied by local DNA demethylation [43]. For each cancer type, we considered the emCpGs lying in open chromatin regions and computed spearman correlations between their level of methylation and the level of openness of the regions that contain them ("Materials and methods" section). As expected, we recapitulated the results previously observed [43] with consistent negative correlations between chromatin accessibility and DNA methylation at emCpGs (Additional file 3: Fig. S6). Although the number of matching patient IDs for the other cancer types investigated is too small, we still observed similar correlation trends.

Taken together, these results provide complementary supporting evidence for the enrichment of emTFs with pioneer function to promote chromatin accessibility and demethylation in a binding site-directed fashion in cancer patients.

### De novo methylation-protected CpGs and CpGs associated with emTFs harbour distinct TF binding signatures

In the previous sections, we revealed that regions around emTF binding sites harboured significant proportions of emCpGs. Nevertheless, not all CpGs proximal to the corresponding TFBSs exhibited DNA methylation levels correlating with the emTFs’ expression across patients. We investigated whether distinct TF binding patterns could discriminate between these two sets of CpGs (correlated/emCpGs versus uncorrelated for each emTF in each cancer type). For each emTF-cancer type pair, we looked for the differential enrichment of TFBSs for 231 TFs using the UniBind enrichment tool [31], when considering regions surrounding emCpGs versus non-correlated CpGs and vice versa ("Materials and methods" section).

**Fig. 2:**
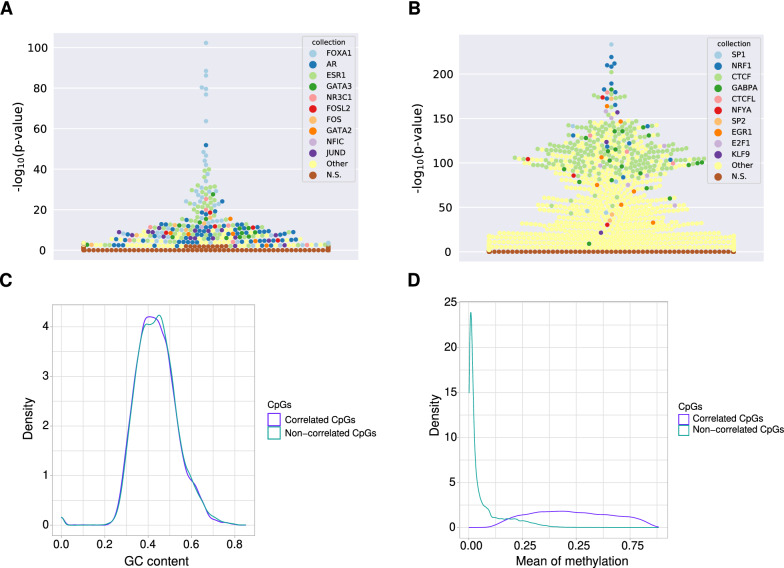
TF binding signatures at FOXA1-associated emCpGs versus de novo methylation-protected CpGs in breast cancer. **A** Beeswarm plot depicting TFBS sets enrichment (*y*-axis) specific to regions surrounding emCpGs associated with FOXA1. Each point corresponds to a TFBS data set in UniBind (one colour per TF, see legend). **B** Beeswarm plot depicting TFBS sets enrichment (*y*-axis) specific to regions surrounding non-correlated CpGs, which are close to FOXA1 TFBSs but whose DNA methylation levels do not correlate with FOXA1 expression in breast cancer samples. **C** Density distribution (*y*-axis) of GC contents (*x*-axis) at regions surrounding FOXA1 emCpGs (purple) and non-correlated CpGs (green). **D** Density distribution (*y*-axis) of mean methylation levels (*x*-axis) across breast cancer samples for FOXA1 emCpGs (purple) and non-correlated CpGs (green)

We consistently observe that regions of ± 200 bp surrounding emCpGs for a given emTF are differentially enriched for binding sites bound by that particular emTF (Fig. 2A and Additional file 1: Data S1). It is important to note that both emCpGs and non-correlated CpGs are close to TFBSs for the given emTF and the regions analysed did not exhibit distinct %GC content (Fig. 2C, Additional file 3: Figs. S7A–S17A). Hence, the differential enrichment analysis highlights that regions flanking emCpGs contain significantly more TFBSs for the emTF than regions flanking non-correlated CpGs, without an overall nucleotide composition difference. Figure 2A depicts a representative example using flanking regions of CpGs close to FOXA1 TFBSs with emCpGs and non-correlated CpGs identified in the BRCA cohort. Note the combined enrichment for FOXA1, ESR1, and GATA3 TFs close to the emCpGs; these 3 TFs have already been associated with DNA methylation patterns in estrogen receptor positive breast cancers [8].

 The analyses of regions surrounding non-correlated CpGs consistently revealed the differential enrichment for TFBSs associated with the TFs CTCF, YY1, NRF1, GABPA, KLF9, and SP1 (Fig. 2B and Additional file 2: Data S2). The enrichment of these TFs is in agreement with previous studies that identified the binding of SP1, CTCF, NRF1, and YY1 to prevent de novo methylation [7, 45–47]. The protective effect of these TFs against de novo methylation is in line with the constant hypomethylation of the non-correlated CpGs observed across emTFs and cancer cohorts (Fig. 2D and Additional file 3: Figs. S7B–S17B).

Altogether, these results support the existence of two distinct TF binding signatures that discriminate emCpGs associated with emTFs from other CpGs close to the TFBSs of emTFs. While the emCpGs harbour enriched binding sites for their specific emTFs, the non-correlated CpGs shared a binding signatures for SP1, CTCF, NRF1, GABPA, KLF9, and YY1 providing a protective effect against de novo methylation across cancer types.

### emCpGs are predicted to regulate genes involved in immune response, cell fate determination, and cancer pathways

**Fig. 3:**
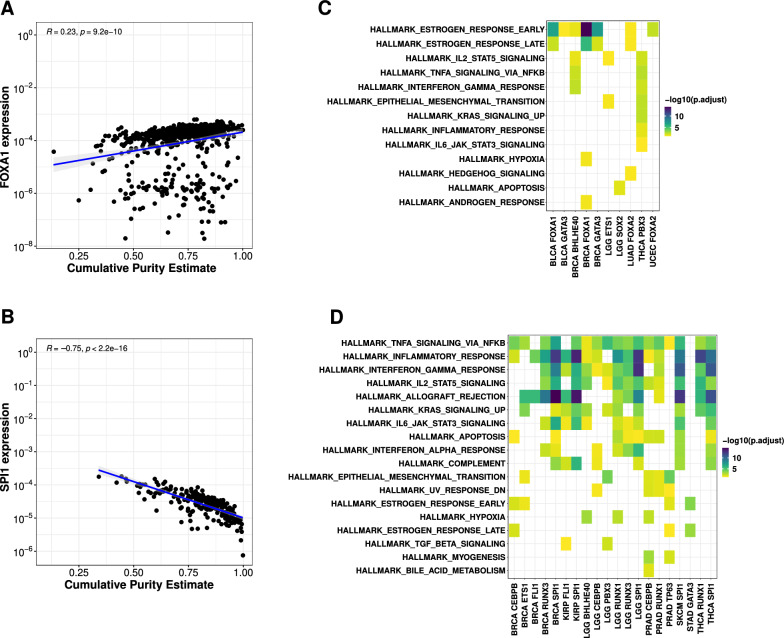
Functional evaluation of the emCpG gene targets. **A** Pearson correlation between FOXA1 expression and tumour purity in BRCA patients. **B** Pearson correlation between SPI1 expression and tumour purity in KIRP patients. As a tumour purity variable, we used cumulative purity estimates from BRCA and KIRP patients, respectively, reported by Aran et al. [52]. The scatterplots compare the tumour purity (*x*-axis; cumulative purity estimate) and expression of the TFs (*y*-axis). The blue lines represent the fitted Pearson linear relationship with the grey zone representing the 95% confidence interval (Pearson R coefficients and associated *p* values are provided in the top-left corner). The expression of FOXA1 in breast cancer patients shows positive correlation indicating that the signals observed in the GO term and pathway enrichments are coming from the tumour cells themselves. The expression of SPI1 in kidney renal papillary cell carcinoma patients shows negative correlation indicating that the signals observed in the GO term and pathway enrichments may be coming from the tumour microenvironment. **C **Functional enrichment analysis for genes linked to emCpGs associated with cancer cell emTFs (i.e. emTFs whose expression positively correlate with tumour purity as in A.). **D** Functional enrichment analysis for genes linked to emCpGs associated with immune cell emTFs (i.e. emTFs whose expression negatively correlates with tumour purity as in B.). Functional enrichments in C–D were performed using the Hallmark sets from MSigDB [49]

With multiple lines of evidence supporting the pioneer function of the emTFs, we hypothesized that they might be involved in the activation of specific genes via demethylation of emCpGs in cis-regulatory regions. As the methylation and expression data from TCGA were derived from bulk tumours, the samples are a combination of cancer cells and cells from the tumour microenvironment. Hence, some emTFs might be acting upon cancer cells, while others would be active in cells from the microenvironment. We investigated the association between the observed emQTL signals and tumour purity of the TCGA samples. By comparing the level of expression of the emTFs with the tumour purity estimate of the samples in the cancer cohorts, we observed a positive correlation for about half of the emTFs ("Materials and methods" section; Fig. 3A, Additional file 3: Fig. S7C–S17C). emTFs BHLHE40, ETS1, FOXA1, FOXA2, GATA3, PBX3, TP63, and SOX2 lie in this category across several cancer types (Fig. 3A, Additional file 3: Fig. S7C–S17C). The positive correlation points to the emQTL signal being mostly driven by cancer cells in the associated cohorts. On the contrary, the expression of some emTFs in specific cancer types was negatively correlated with tumour purity (Fig. 3B, Additional file 3: Fig. S7C–S17C). emTFs CEBPB, ETS1, FLI1, BHLHE40, TP63, GATA3, PBX3, RUNX1, RUNX3, and SPI1 lie in this category across several cancer types (Additional file 3: Figs. S7C–S17C). The negative association with tumour purity indicates that these emTFs might be acting in cells from the microenvironment in the corresponding cancer types.

To assess the functional relevance of the identified emCpGs in these different cellular contexts, we estimated the enrichment for biological processes and pathways in the list of genes linked to emCpGs for each pair of emTF-cancer types. We linked emCpGs to genes using gene-specific regulatory elements defined by the STITCHIT algorithm, which relies on an integrative analysis of epigenetic and transcriptomic data [48]. This method allows to assign emCpGs lying in distal *cis*-regulatory elements to their potential target genes. When emCpGs did not lie within STITCHIT regulatory elements, we assigned them to the closest gene ("Materials and methods" section). The number and proportion of emCpGs in each pair of emTF-cancer type linked to genes using the STITCHIT method versus the distance-based method are provided in Additional file 4: Table S6. The emCpG-gene links were derived from multiple cell types/tissues but we aimed to focus on the most likely regulatory links in a cancer type-specific way. Specifically, we required a significant (Bonferroni adjusted *p* value < 0.01) negative correlation between emCpG methylation level and target gene expression in a given cancer type to conserve an emCpG-to-gene link.

The genes linked to emCpGs associated with cancer-cell emTFs were mostly found enriched in hormone- and cancer-associated Hallmark sets of genes from the Molecular Signatures Database [49] (MSigDB; Fig. 3C). For instance, emCpGs associated with FOXA1, FOXA2, and GATA3 were linked to genes enriched in estrogen receptor signalling pathways; SOX2 emCpGs enriched for genes associated with apoptosis; ETS1 emCpGs enriched for genes associated with epithelial to mesenchymal transition (Fig. 3C). Moreover, we observed the recurrent enrichment for genes in Gene Ontology biological processes (GO-BP) associated with cell fate determination and development (i.e. differentiation-, development-, morphogenesis-, and growth-related terms; Additional file 3: Fig. S18A). The enrichment for these processes is in line with the biological function of pioneer TFs, which are associated with the control of cell fate and cell lineage reprogramming in normal development and cancers [16, 33, 50, 51].

When considering emCpGs linked to emTFs associated with cells from the tumour microenvironment, we observed the recurrent functional enrichment for immune-related terms both from MSigDB and GO-BP (Fig. 3D, Additional file 3: Fig. S18B). The functional enrichment observed suggests that the emQTL signal associated with these emTFs in the corresponding cancer cohorts is derived from tumour infiltrating lymphocytes.

Taken together, these results highlight that some emTFs are likely associated with immune cells in the tumour microenvironment, while other emTFs are likely driving local demethylation of targeted cis-regulatory regions.

### Experimental assessment of the impact of FOXA1 expression on DNA methylation in MCF-7 breast cancer cells

We sought to experimentally assess the impact of the expression of an emTF on DNA methylation around its TFBSs using a cancer cell line. We selected FOXA1 and evaluated the impact of its expression in the MCF-7 breast cancer cell line. Specifically, we profiled DNA methylation in MCF-7 cells using Illumina EPIC methylation arrays under three conditions in triplicate: (1) control, (2) endogenous knock-down (KD) of FOXA1, and (3) rescue of the endogenous KD by transient ectopic expression of FOXA1-V5 (see Additional file 3: Fig. S19 for evaluation of the KD and transient rescue efficiencies using western blot). Compared to the control condition, the KD experiment assessed DNA methylation with less FOXA1 proteins, while the transient ectopic expression of FOXA1-V5 was used to try to rescue endogenous expression of FOXA1 after KD and to evaluate how it could restore the DNA methylation phenotype observed in the control condition.

**Fig. 4:**
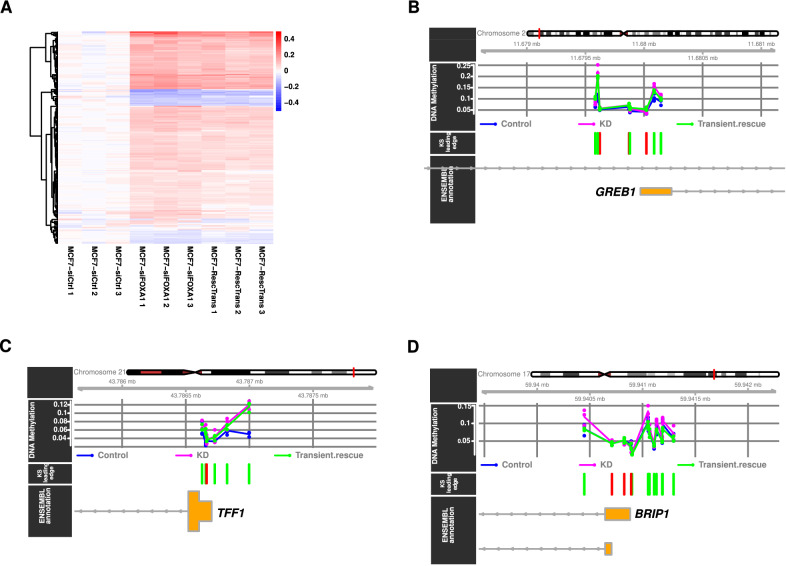
FOXA1 KD in MCF-7 cells leads to local DNA methylation increase. **A** Heatmap depicting DNA methylation β-values at 228 CpGs (rows) in DMRs (the mean β-value at the control replicates are subtracted to the β-value of each CpG, see "Materials and methods" section). Blue indicates demethylation when compared to the control replicates and red indicates increased methylation. See Additional file 3: Fig. S19 for immunoblotting evaluation of the three conditions (control, KD, and rescue). **B**, **C**, **D** Genomic context and methylation information at 3 of the 229 identified DMRs, which correspond to the promoter regions of the *GREB1* (B), *TFF1* (C), and *BRIP1* (D) genes. The upper panels provide the location in the corresponding chromosome. The second panel from the top provides the beta-values at the CpGs in the regions (blue for control samples, purple for knock-down (KD), and green for transient rescue). The third panel from the top indicates in green the significant CpGs used to predict the DMR, while the non-significant CpGs are depicted in red

We specifically evaluated the effect of FOXA1 KD on DNA methylation at genomic regions observed to be bound by FOXA1 in MCF-7 cells captured by ChIP-seq experiments ("Materials and methods" section). DNA methylation levels of the 83,521 CpGs within FOXA1 ChIP-seq peak regions were compared between control and KD replicates with the mCSEA tool [53] to identify differentially methylated regions (DMRs; see "Materials and methods" section). mCSEA predicted 229 DMRs (adjusted *p* value < 0.1), encompassing 431 CpGs. We observed that CpGs within the DMRs mostly exhibited higher levels of methylation after FOXA1 KD (Fig. 4A). Rescuing FOXA1 expression using transient ectopic expression of FOXA1-V5 did not restore methylation at the identified DMRs after 24 h (Fig. 4A). The lack of demethylation observed after 24 h of ectopic expression of FOXA1-V5 might be due to a slow DNA methylation process as previously observed [13]. Figure 4B–D provides case examples of DMRs after FOXA1 KD in the promoter regions of genes that have previously been associated with breast cancer: *GREB1* (growth regulation by estrogen in breast cancer 1, a regulator of hormone-dependent breast cancer growth [54]), *TFF1* (trefoil factor 1, an estrogen-regulated protein [55]), and *BRIP1* (BRCA1 Interacting Protein C-Terminal Helicase 1, whose mutants participate in breast cancer development [56]).

The experimental results outlined here confirm the association between FOXA1 expression and DNA methylation levels at genomic regions bound by FOXA1. The KD of FOXA1 increased methylation at regions bound in MCF-7 by FOXA1, supporting the link between FOXA1 binding and local demethylation.

### FOXA1 physically interacts with TET1 and TET2 at endogenous levels

The observations above suggest that FOXA1 is associated with demethylation, which can be achieved by the TET1 and/or TET2 proteins. While FOXA1 has been shown to interact with TET1 in the LNCaP (lymph node carcinoma of the prostate) cell line [23], no interaction has been reported in breast cancer cell lines with neither TET1 nor TET2, to the best of our knowledge. We aimed to assess potential protein–protein interactions between FOXA1 and TET1 and/or TET2 in the MCF-7 cell line.

**Fig. 5:**
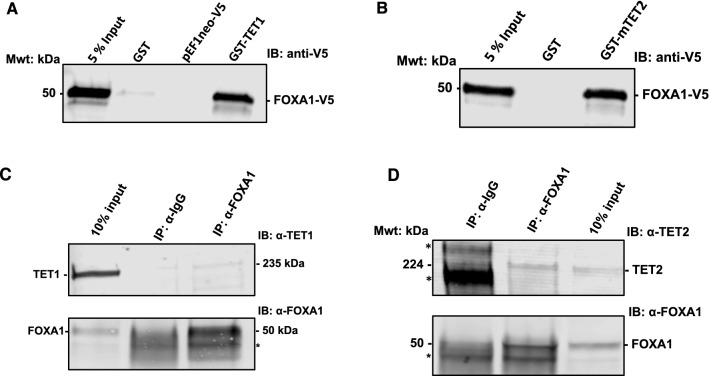
FOXA1 endogenous interaction with TET1 and TET2. We employed endogenous IP on nuclear extracts obtained from MCF-7 cells. **A** GST-pulldown of FOXA1-V5 using GST-TET1. **B** GST-pulldown of FOXA1-V5 using GST-mTET2. **C** Immunoprecipitation of endogenous FOXA1 using rabbit anti-FOXA1 antibody to detect endogenous TET1 pulled together with endogenous FOXA1. **D** Immunoprecipitation of endogenous FOXA1 using rabbit anti-FOXA1 antibody and detecting endogenous TET2 pulled together with FOXA1. Non-specific bands are marked with asterisks (*)

We first assessed interactions for FOXA1 with TET1 and TET2 in vitro through GST-pulldown assays. The assays were performed using N-terminally GST fused full length human TET1 and mouse TET2 isoform 2 (mTET2) ("Materials and methods" section). Note that the mTET2 aligns well with the C-terminal half of the human TET2 (from residue 1388 to 2002, see Additional file 3: Fig. S20A for protein sequence alignment and Additional file 3: Fig. S20B for structural alignments of hTET2 and mTET2). Using the GST tagged TET proteins, we successfully pulled out FOXA1-V5 from COS-1 cells whole protein extract, where FOXA1-V5 was transiently transfected in these cells for 24 h (Fig. 5A, B).

Next, we investigated the interactions between FOXA1 and the TETs in vivo in MCF-7 cells. We performed endogenous immunoprecipitations using nuclear extracts derived from MCF-7 cells ("Materials and methods" section). Immunoprecipitation of FOXA1 revealed an interaction with TET1 and TET2 endogenously at physiological levels in MCF-7 cells (Fig. 5C, D).

Taken together, these results suggest that TET1 and TET2 interact with FOXA1 and that they are recruited by FOXA1 in MCF-7 cells for local demethylation. These interactions further support the in silico predictions for the importance of FOXA1 in driving local demethylation patterns in breast cancer.

## Discussion

We established a computational framework that allowed for a systematic investigation of the interplay between TF binding and DNA methylation in cancer patient samples. Through emQTL computations, we predicted 13 TFs to be associated with DNA methylation patterns around their binding sites across several cancer types. We confirmed that specific genomic regions are protected against de novo methylation and harbour a characteristic TF binding motif signature with enrichment of binding sites for SP1, CTCF, NRF1, GABPA, KLF9, and/or YY1. The 13 emTFs are strongly enriched for TFs with previously established pioneer function, which enables them to engage with closed chromatin and reshape the chromatin landscape. We found that some of the emTFs are likely acting in cancer cells, while others are more likely specific to cells from the tumour microenvironment (most probably immune infiltrating cells). Accordingly, the CpGs whose methylation levels are associated with the expression of the emTFs are predicted to regulate genes enriched for cancer-associated or immune pathways. Finally, we experimentally (i) confirmed the effect of FOXA1 expression on DNA methylation patterns at regions bound by FOXA1 in the MCF-7 breast cancer cell line, and (ii) detected interactions of FOXA1 with TET1 and TET2 proteins both in an in vitro setup and at endogenous levels. The in vitro GST-pulldown assay of GST fused mTET2 further revealed that the interaction observed between TET2 and FOXA1 is mapped to the C-terminal end of TET2 (amino acid residues 1388-2002). Altogether, the findings outlined in this study provide evidence supporting the importance of specific pioneer TFs in reshaping the chromatin landscape in cancer patients to rewire gene regulatory networks through local DNA demethylation of *cis*-regulatory regions.

The results highlighted in this report complement previous investigations of the interplay between TF binding and DNA methylation. The high-throughput screening approach developed by Suzuki et al. [24] exhibited that some developmental TFs induce binding site-directed DNA demethylation. The screening approach requires to select a set of TFs to test and is based on TF overexpression in specific cell lines, while the emQTL approach allows for the large-scale assessment of TFs from cancer patient material. The emQTL methodology has the potential to highlight the physiological and spatio-temporal context of TFs’ expression in cancer samples. Other computational studies predicted TFs involved in shaping the methylation landscape of cancer cells [26, 27, 57, 58]. These studies focused on CpGs that were hypo- or hyper-methylated in cancer when compared to healthy samples, while our framework considers the variation of methylation across cancer samples for all CpGs. Moreover, the ELMER and TENET software are restricted to CpGs lying in potential enhancers that need to be identified with orthogonal data relevant to the cell type associated with each cancer type. We show that emCpGs were predominantly located in intronic and intergenic regions, which are often associated with enhancers. This observation combined with previous predictions [26, 27, 57, 58] suggests that the emTFs are likely to drive demethylation at numerous enhancer regions. Nevertheless, we highlighted that ~ 35% of the emCpGs are in promoter regions (Additional file 3: Fig. S4), which cannot be captured by ELMER or TENET. Our approach focuses on DNA methylation patterns focal to TFBSs to predict driver TFs, while other studies rely on TF binding motif enrichment. As often in computational biology, we believe all these approaches are complementary as they address different aspects of the underlying biological mechanism. Among the predicted upstream regulators identified by ELMER while leveraging DNA methylation status at transcriptional enhancers [26], multiple predictions are in agreement with the emTFs identified here: FOXA1 and GATA3 in BRCA, FOXA2 in UCEC, RUNX1 in KIRP, and SOX2 and TP63 in LUSC cohorts. Similarly, both TENET [27] and our study predicted FOXA1, GATA3, ESR1, and SPI1 as emTFs in BRCA and CEBPB and RUNX1 as emTFs in PRAD. In addition, Detilleux *et* *al*. [58] and the present study both identified FOXA1, GATA3, ESR1, CEBPB, and BHLHE40 as potential divers of methylation patterns in BRCA and FOXA2 in UCEC.

We considered in this study a collection of TFBSs with experimental and computational support for direct TF–DNA interactions, which are stored in the UniBind database [29]. This collection was obtained through the uniform processing of thousands of ChIP-seq experiments from diverse cell types/tissues and conditions. We acknowledge that some TFBSs might not be functional in the cancer cells or cell type of origin analysed here. Nevertheless, they provide the necessary background for large-scale analysis and these regions have been identified as TF-bound in biological contexts. Furthermore, the TFBSs stored in UniBind represent evolutionarily conserved elements [29] and harbour similar mutational load than protein-coding exons (using TCGA somatic mutation data), supporting their functional relevance [59].

In the emQTL analysis performed in this study, TF expression was used as a surrogate to the capacity of TFs to bind their TFBSs. We considered ~ 400 bp surrounding TFBSs (± 200 bp) to assess the local effect of TF binding on DNA methylation following [24], where the authors estimated that TF-induced DNA demethylation was local to the TFBSs with a range of a few hundred base pairs (from ~ 106 to ~ 320 bp). We acknowledge that the RNA expression of a TF might not always relate to its capacity to bind its TFBSs. Nevertheless, increasing TF concentration is related to the capacity of a TF to bind more DNA segments with distinct affinities [60]. Furthermore, we acknowledge that the regulatory activity of TFs goes beyond what can be estimated through their level of transcription. Indeed, several post-translational modifications (PTMs), such as phosphorylation, SUMOylation, ubiquitination, acetylation, glycosylation, and methylation are regarded as one type of regulatory mechanism controlling the activity of TFs [61–64]. Unfortunately, capturing PTM information for all TFs in cancer samples is intractable. Past efforts aimed at classifying TFs based on their functional features, such as their involvement in signal response versus cell specific developmental function [65]. It is noteworthy that several of the 13 emTF appear to be in the developmental group. For instance, GATA3 is required for the T-helper 2 (Th2) differentiation process (reviewed in [66]); C/EBPB in adipocyte differentiation [67, 68]; PBX3 is a homeodomain protein, which are known to be important for human developmental processes [69]; RUNX1 and RUNX3 have a primary role in the development of all hematopoietic cell types [70]; and FLI1 plays an essential role in embryogenesis, vascular development, and megakaryopoiesis [71, 72]. As expected, this may indicate that our methodology selects for TFs whose expressions are of key importance for their function.

We further recognize that, as we previously observed for emQTLs in general [8], emTFs are not specific to cancer cells. Indeed, TCGA data were obtained from populations of heterogeneous cancer cells and cells from the tumour microenvironment. Nevertheless, we argue that the heterogeneity of the cells provides the appropriate means to perform correlation analysis, such as emQTLs. Furthermore, this strategy provided us with the opportunity to capture signals coming from both cancer cells and immune cells, which could be disentangled through the assessment of tumour purity in TCGA samples.

Several studies previously proposed a model where pioneer TFs remodel the chromatin landscape through increased accessibility followed by DNA methylation loss priming inaccessible enhancers during cell fate transitions (reviewed in [19]). Barnett et al*.* validated this model by profiling DNA methylation and chromatin accessibility at the same time from a single DNA fragment, where they differentiate THP-1 cells into naive M(-) macrophages. They reported that along the enhancer regulation continuum during differentiation of THP-1 cells, loss of DNA methylation is necessary for cell fate determination [19]. Similarly, Reizel et al. demonstrated that FOXA1 and FOXA2 TFs are responsible for DNA demethylation at tissue-specific enhancers during liver development, likely through the recruitment of TET2/3 enzymes [73]. Furthermore, pioneer TFs act as developmental factors by controlling key regulatory processes leading to cell identity changes. With the predicted emTFs strongly enriched for pioneer function, we hypothesize that they trigger the aberrant activation of developmental cis-regulatory regions leading to cell identity transitions during carcinogenesis. This hypothesis is in agreement with previous observations of architectural protein- and pioneer TF-mediated chromatin rearrangements that lead to reactivation of embryonic gene expression signatures occurring during cancer (reviewed in [34]).

Several TFs have been reported with a protective role against de novo DNA methylation. These TFs include SPI1 [45, 46, 74–76], YY1, NRF1 [7, 75], GABPA, NF-YA [75], CTCF [47], and KLF9 [77]. In line with these reports, we found consistent enrichment for TFBSs associated with these TFs proximal to CpGs harbouring constant hypomethylation across patients, despite the presence of TFBSs for emTFs. On the contrary, regions surrounding emCpGs were enriched for TFBSs associated with the emTFs. This enrichment suggests that several TFBSs for the same emTF colocalize in regulatory regions, which is a signature of homotypic clusters of TFBSs [78]. These homotypic clusters have been described as key components of human promoters and enhancers and have been found to be enriched in developmental enhancers [78]. The expression of the majority of the emTFs exhibited anti-correlation with CpG methylation close to their TFBSs, indicating that these emTFs are likely inducing local DNA demethylation. This is in agreement with previous studies that reported RUNX1 [22, 79], RUNX3 [24], SPI1 [21, 24, 79], BHLHE40 [79], and FOXA1 [25] to induce binding-site directed DNA demethylation. Altogether, these observations provide further evidence for the involvement of emTFs in the specific transcriptional activation of developmental cis-regulatory regions in cancers.

We also captured positively correlated emCpGs, wherein higher TF expression is associated with higher DNA methylation near their TFBS, although in smaller proportions. It is noteworthy that recent work from diverse model systems suggests that 5mC might not always act as a dominant repressive mechanism and that hypermethylated promoters and enhancers can be permissive to transcription in vivo and in vitro (Reviewed in [80]).

The emQTL analysis did not examine specifically the CpGs lying within TFBSs but rather considered CpGs located at most 200 bp regions away from the TFBSs. As a consequence, the impact of DNA methylation at the TFBSs was not specifically addressed. From the 13 emTFs predicted, eight have either previously been shown in vitro to prefer binding methylated sites [81] or recognize binding motifs that do not contain CpGs (GATA3, SOX2, PBX3, CEBPB, FOXA1, FOXA2, SPI1, and TP63). These characteristics provide an advantage for these TFs to act as pioneer factors, since their binding would not be precluded by methylation in closed chromatin regions. On the contrary, in vitro evidence suggests that the five remaining TFs (ETS1, BHLHE40, FLI1, RUNX1, and RUNX3) do not bind, or more weakly, to methylated sites [81]. Nevertheless, the inhibition of binding via DNA methylation detected in vitro is not always observed in vivo or can be restricted to some genomic regions [82–84]. Supporting evidence for pioneer function has been reported for FLI1 [40, 85, 86], RUNX1, and RUNX3 [87, 88]. How the rest of the remaining factors can engage with closed chromatin would require further investigations.

We associated emCpGs with target genes by relying on (i) the STITCHIT database of regulatory elements to gene links [48] or (ii) genomic distance. It is well known that cis-regulatory elements may regulate distal genes, which are not necessarily the closest ones [89]. By prioritising regulatory elements to gene links from STITCHIT, we aimed to rely on regulatory associations previously observed in a large collection of cell types. As some links between regulatory elements to genes might be false positives and as some links might be cell type-specific, we exclusively kept the CpG-gene pairs exhibiting anti-correlation (between DNA methylation and expression) to refine the associations in a cancer type-specific way.

The functional enrichment analyses for the genes predicted to be targets of emCpGs confirmed that the emQTL signals were likely derived from either cancer cells or tumour-infiltrating cells. Indeed, bulk tumour samples from TCGA that were analysed in this study represent a mixture of cancer cells and cells from the tumour microenvironment [52]. The correlation between emTF expression and tumor purity in the samples allows for the discrimination between the two types of signals. However, the heterogeneity of cancer cells, which belong to several clonal populations, provides an additional level of complexity that was not considered in this study. Nevertheless, the identified emTFs are likely to play a major role in shaping the chromatin landscape at cis-regulatory regions controlling the transcription of cancer- or immune-related genes, respectively.

The experimental assessment of the effect of FOXA1 expression on DNA methylation in the MCF-7 breast cancer cell line revealed a limited number of FOXA1-bound regions with significant differential methylation. This is in line with a recent study [58], where CRISPR knockout (KO) of FOXA1 or GATA3 in HCC1954 cells followed by whole genome bisulfite sequencing revealed 84 FOXA1 hypermethylated regions around FOXA1 TFBSs and 30 around GATA3 TFBSs. The limited effect detected could be explained by the fact that DNA methylation is a stable epigenetic mark and the dynamic regulation of methylation and demethylation are rather slow processes. Indeed, the investigation of DNA methylation turnover using experimental and theoretical frameworks revealed that it takes from several days to weeks [13]. Furthermore, the efficiency of transient transfection of the FOXA1 expression plasmid might be lower than the siRNA transfection efficiency, which can contribute to the small effect observed. As our experimental setup subjected the cells to siRNA-mediated KD for 72 h and to transient rescue of FOXA1-V5 ectopic expression for 24 h, some longer term effects have been missed.

In summary, we reported an interplay between TF binding and DNA methylation marks, where the binding of pioneer TFs at their TFBSs are likely to trigger local DNA demethylation that could lead to carcinogenesis. These results confirm the central role for pioneer TFs in aberrant DNA demethylation patterns in cancers. While we experimentally assessed the effect of a single TF in a cell line, the predictions outlined in this report could be followed up through experimental validation to assess their capacity to drive methylation patterns and carcinogenesis.

## Materials and methods

### TCGA RNA-seq and methylation data

We obtained patient RNA-seq and DNA methylation array (Illumina 450 K arrays) data collected by TCGA for 19 cancer types (LAML-US, BRCA-US, PRAD-US, LUAD-US, LUSC-US, COAD-US, LIHC-US, HNSC-US, THCA-US, GBM-US, LGG-US, KIRC-US, KIRP-US, UCEC-US, STAD-US, SKCM-US, PAAD-US, CESC-US, and BLCA-US) from the ICGC data portal [28, 90]. The number of samples for which both RNA-seq and DNA methylation array data was available is provided in Additional file 4: Table S1.

### Transcription factor binding sites

Direct TF–DNA interaction predictions were retrieved from the UniBind database (version 2018) for 231 human TFs [29]. TFBS coordinates were provided using the GRCh38 assembly of the human genome and were converted to the GRCh19 assembly using the UCSC liftOver tool [91].

### emQTL computation

We performed emQTL analyses by computing Spearman correlations between the levels of methylation at CpGs and TF expression levels in each cohort independently using the same methodology as previously described [8] with the eMap R package (version 1.2) [92]. The emQTL computation was restricted to CpGs at most 200 bp away from UniBind TFBSs. Intersections between CpG coordinates and extended TFBS regions were obtained using the BedTools version 2.26.0 [93]. For each cancer type, we only considered CpGs with an interquartile range of methylation beta values > 0.1 for the computation as in [8].

For each TF in each cancer type, we selected the correlated CpGs with a Bonferroni corrected *p* value < 0.01. We only further considered TFs significantly correlated with at least 5000 CpGs for downstream analyses. To assess the enrichment for correlated CpGs close to the TF’s TFBSs, we performed Mann–Whitney *U* (MWU) tests with the set of considered CpGs in the corresponding cohort as the universe. TF-cancer type pairs were considered significant with a MWU Bonferroni-corrected *p* value < 0.01. An overview of the computational workflow is provided in Additional file 3: Fig. S1A.

### Upset and venn diagram plots

All upset and Venn diagram plots were obtained using Intervene (version 0.6.4) [94].

### Pioneer and flanking accessibility-associated TFs

We compiled a list of pioneer TFs from the literature [33–42] (Additional file 4: Table S4). The list of flanking accessibility-associated TFs were retrieved from [43], where they have been described to be associated with increased flanking accessibility around their motif centre in cancer samples from ATAC-seq data. We considered in our study the 29 flanking accessibility-associated TFs that were tested for emQTL in this report (Additional file 4: Table S5). We assessed the significance of the intersection between the list of pioneer TFs (or the list of flanking accessibility-associated TFs) and the emTFs by performing Fisher tests with the Bioconductor GeneOverlap package (version 1.18.0) [95].

### Comparison between emCpGs and non-correlated CpGs

For each emTF-cancer type pair, we assessed the enrichment for TFBSs around the corresponding emCpGs and non-correlated CpGs. We computed differential enrichment of TFBSs between regions of ± 200 bp centred around the emCpGs versus the non-correlated CpGs, and vice versa. Genomic regions were lifted, using the liftOver tool from UCSC [91], from the GRCh19 genome assembly over to the GRCh38 version, which is the assembly used in UniBind. Differential enrichment of TFBS sets was performed using the *twoSets* subcommand of the UniBind enrichment tool (https://unibind.uio.no/enrichment/; https://bitbucket.org/CBGR/unibind_enrichment/) using the collection of TFBS sets from UniBind version 2018 [29, 31]. Specifically, the foreground set of regions corresponded to the regions centred around emCpGs or non-correlated CpGs and the combined set of such regions was used as background.

The %GC distributions at genomic regions centred around emCpGs and non-correlated CpGs were computed by the BedTools *nuc* function.

### Association between emCpGs and target genes

We downloaded the associations between regulatory elements and target genes predicted by STITCHIT from the ENCODE, Roadmap, and Blueprint data sets at https://zenodo.org/record/2547384#.XIK0x-RYZ14. The coordinates of the emCpGs considered for each emTF in each cohort were lifted from GRCh19 over to GRCh38 coordinates and intersected with STITCHIT regulatory elements using the *intersect* subcommand of the BedTools. CpGs lying within the regulatory elements were associated with the corresponding target genes. The CpGs not overlapping with STITCHIT regulatory elements were linked to genes with the nearest TSS using the HOMER *annotatePeaks.pl* script [96].

### Genomic distribution of CpGs

We used the *annotatePeaks.pl* script from HOMER [96] to compute the genomic distribution of all CpGs investigated (n = 376,997) and of the emCpGs in each cancer type (Additional file 3: Fig. S4).

### Correlation between ATAC signal and methylation at emCpGs

We downloaded the TCGA ATAC-seq data described in [43] from https://gdc.cancer.gov/about-data/publications/ATACseq-AWG. We considered the cancer cohorts with at least 20 samples for which DNA methylation was available in our study. We selected the emCpGs predicted in each cancer type and their surrounding ± 200 bp regions and intersected them with the pancancer ATAC-seq peaks provided in [43] using the BedTools *intersect* subcommand. Finally, spearman correlation between the level of methylation at emCpGs and the level of ATAC-seq normalised counts at the underlying peaks were computed in each cancer type.

### Functional enrichment analysis

Genes associated to emTFs in cancer cohorts were submitted to the *clusterProfiler* R package (version 3.12.0) [97] to compute enrichment for gene ontology (GO) biological processes and MSigDB Hallmark sets (the *gmt* file corresponding to the Hallmark set was retrieved from MSigDB v7.4). Redundant enriched GO terms were reduced using the *GOSemSim* R package (version 2.10.0) [98]. For MsigDB Hallmark set enriched terms, we considered the top 10 enriched terms (ranked by Benjamini and Hochberg adjusted *p* values < 0.05) per emTF-cancer pairs for drawing the figures. In Additional file 3: Fig. S18, we considered the GO terms with Benjamini and Hochberg adjusted *p* values < 0.05 and plotted the top 5 most enriched terms per emTF-cancer pair. Enrichment plots were produced using the *geom_tile* function from the *ggplot2* R package (version 3.3.3).

### Tumour purity

We downloaded cumulative tumour purity estimates from [52]. For the STAD-US cohort, the cumulative tumour purity was not computed in [52]; we retrieved tumour purity scores for STAD-US samples from the ICGC data portal (dcc.icgc.org/releases/PCAWG/consensus_cnv). Pearson correlations between tumour purity and TF RNA expression were computed using the *stat_cor* R function with the parameter *method* = *”pearson”* using the *ggscatter* function in *ggplot2*.

### Bioinformatics analysis of mTET2 and hTET2 proteins

To assess the interaction between TET2 and FOXA1, we used mTET2 GST fusion protein. To assess the relevance of using mTET2 in the GST pull down assay, we assessed the amino acid sequence conservation between mTET2 isoform 2 (RefSeq ID: NP_001035490) and the human TET2 (hTET2; RefSeq ID: NP_001120680). We visualised the pairwise sequence alignment of the two proteins using the MUSCLE algorithm accessed through Jalview (version 2.11.1.4) [99]. It revealed that mTET2 aligns well with the C-terminal half of hTET2 (pairwise sequence identity = 59.72%; Additional file 3: Fig. S20A). To further highlight the conservation of the TET2 proteins between mouse and human at the structural level, we obtained the Protein Data Bank structures corresponding to hTET2 (PDB ID: 4nm6A) and the modelled structure of mTET2 (PDB ID: Q6NO21). We compared the two structures with the *ce align* algorithm implemented in pyMOL version 2.4.2, which is represented in Additional file 3: Fig. S20B.

### Plasmid construction

The human FOXA1 sequence with RefSeq accession ID NM_004496 was synthesised with a C-terminal V5-tag sequence and obtained in pCIneo vector with NheI and XhoI cloning sites from GenScript. The sequence with the C-terminal V5-tag was transferred to the pEF1neo mammalian expression vector using NheI and SalI. pEF1neo is a vector generated from pCIneo by replacing the CMV promoter with the human EF1-alpha promoter. It generates a mammalian expression vector for FOXA1 as pEF1neo-FOXA1-V5. GST-TET1 fusion protein was made by transferring the full length TET1 sequence into the pGEX-KG vector, which was derived from pGEX-2 T as described in [100]. The pGEX-KG vector was first cut with XmaI and was filled in with Klenow (Roche Applied Science) to form blunt end, this was followed by XbaI digestion. N-terminally FLAG- and HA-tagged TET1 from the mammalian expression plasmid pEF1-FH-TET1 (ABCAM) was digested with BmgBI (blunt end cutter) and XbaI. The 6436 bp fragment was then inserted into the XmaI and XbaI digested pGEX-KG vector. *Mus musculus* TET2 (mTET2) clone IMAGE ID 4,977,050 was obtained from Source Bioscience and PCR cloned using oligos (Tet2fwd: 5ʹ-GGGGACAAGTTTGTACAAAAAAGCAGGCTTAatgccaaatggcagtacagt-3ʹ and Tet2rev: 5ʹ-GGGGACCACTTTGTACAAGAAAGCTGGGTTtcatacaaatgtgttgtaag-3ʹ) into pDonor221 (Invitrogen Gateway, ThermoFisher) and sequenced. mTET2 was then cloned into pGEX-AB-GAW by LR reaction for recombinant protein expression of GST fused mTET2.

### Cell cultures and siRNA and plasmid transfections

MCF-7 cells (ATCC^®^ HTB-22™ *Homo sapiens*, epithelial, mammary gland, breast; derived from metastatic site: pleural effusion, adenocarcinoma) were maintained in RPMI-1640-GlutaMAX supplement medium supplemented with 10% FCS (foetal calf serum) and 1% PS (penicillin/streptomycin), and were grown at 37 °C and 5% CO_2_.

We performed siRNA mediated KD of endogenous FOXA1 from MCF-7 cells using custom synthesised siFOXA1 from Qiagen. The siRNA sequences that target the 3ʹ-UTR of FOXA1 were described in [101]. For control transfections, we used the AllStars Negative Control siRNA (Cat.No 1027281, Qiagen). Both siCtrl and siFOXA1 at a concentration of 10 µM were delivered to cells using the lullaby siRNA transfection reagent (OZ biosciences). Specifically, cells were seeded at a density of 10^5^ cells in 6 well plates 24 h prior to siRNA transfection. The next day, the media was changed and siRNAs were delivered using lullaby siRNA transfection reagent. The cells were subjected to siRNA mediated KD for 72 h at 37 °C and 5% CO_2_ before they were harvested for DNA isolation. Transient rescue of the endogenous KD was made 2 days post-siRNA transfection by delivering 2.5 µg of pEF1neo-FOXA1-V5 plasmid with the lullaby transfection reagent for 24 h.

For GST pull-down assay, COS-1 cells were transiently transfected with either 5 or 10 μg of each of pCIneo-FOXA1-V5 and pCIneo-V5 plasmids using lipofectamine 3000 Reagent (Invitrogen).

### Methylation array profiling in MCF-7 cells and bioinformatics analysis

Genomic DNA from MCF-7 cells transfected with either siCtrl or siFOXA1 and MCF-7 cells subjected to siFOXA1 mediated endogenous KD and rescue with exogenous FOXA1-V5 in three biological replicates was isolated using NucleoSpin^®^ Tissue genomic DNA isolation kit (Machery-Nagel). From each sample, 45 µl genomic DNA amounting to 500 ng concentration was delivered to the Genomics core facility at Oslo University hospital, where EPIC array profiling was performed. Bisulfite-converted DNA was amplified, fragmented, and hybridised to Illumina Infinium Human Methylation 850 K Beadchip using standard Illumina protocol.

EPIC array methylation data in IDAT format were normalised with the *minfi* (version 1.36.0) R package [102] using the within array Noob function followed by quantile normalisation as recommended by shinyÉpico [103]. *M* values were obtained from the normalised β-values using *minfi*. Contrasts of *M* values were computed using the *limma* (version 3.46.0) R package between control and KD replicates with the *limma::arrayWeights* option to mitigate the influence of the arrays. The computed raw *p* values from the *limma* fit were provided to the *mCSEATest* function of the *mCSEA* R package (version 1.10.0) to compute differentially methylated regions considering FOXA1 ChIP-seq peaks. FOXA1 ChIP-seq peaks were retrieved from the ReMap 2020 database [104] considering ChIP-seq experiments performed in MCF-7 cells without target or biotype modification.

To draw the heatmap provided in Fig. 4A, we considered all CpGs in the EPIC array lying within the identified DMRs (*n* = 431). For each CpG, we computed the average of the β-values across the three control replicates. The average value was subtracted from the β-value of each CpG in each of the nine samples. Finally, we filtered out the resulting values *vals* that satisfied − 0.05 < *vals* < 0.05. The remaining values associated with 228 CpGs were plotted in Fig. 4A using the *pheatmap* R package (version 1.0.12).

### MCF-7 nuclear extract preparation

Nuclear extracts from MCF-7 cells were prepared as described in [105] with a slight modification. To disrupt the cytoplasmic membrane, in addition to douncing, detergent was used by supplementing buffer A with 0.05% NP-40.

### Antibodies

For western blot (WB) validation of positive transfections and endogenous KDs, we used the following primary antibodies: mouse anti-V5 monoclonal antibody (46-0705, Invitrogen), rabbit anti-FOXA1 M2 polyclonal antibody (GTX100308, Gene-Tex), and mouse anti GAPDH monoclonal antibody (AM4300, Invitrogen). We used the following secondary antibodies for WB: anti-mouse IRDye^®^ 680 RD (925-68072, LICOR) and anti-mouse IRDye 800 CW (925-32213, LI COR).

For endogenous immunoprecipitation, we used the following antibodies: anti-FOXA1 M2 rabbit polyclonal antibody (GTX100308, Gene-Tex), anti-TET1 mouse monoclonal antibody (GTX627420, Gene-Tex), anti-TET2 Rabbit monoclonal antibody (D6B9Y, cell signalling technologies), normal rabbit IgG (2729S, cell signalling technologies), normal mouse IgG (sc-2025, Santa Cruz), and protein G Dynabeads (10004D, Invitrogen).

### GST-pulldown and immunoprecipitation assays

The GST fusion proteins and GST were expressed and isolated as described in [106]. Total cell lysates from COS-1 cells 24 h post transfection were prepared using 300 μl of KAc interaction buffer (Roche Applied Science). GST fusion proteins were bound to glutathione–Sepharose beads (GE Healthcare) by rotating in binding buffer (50 mM Tris HCl pH 8.0, 150 mM NaCl, 5 mM EDTA, 1% Triton X-100, 1 mM Dithiothreitol (DTT) and 1 × Complete protease inhibitor cocktail at 4 °C for 1 h prior to pull-down. The pre-bound fusion proteins were then incubated for 1 h at 4 °C with whole cell lysate obtained from transfected COS-1 cells. The beads were washed 3 × in 500 μl of KAc interaction buffer. The bound proteins were eluted in 40 μl of 3 × SDS loading buffer at 95 °C for 10 min and detected using immunoblotting after SDS‐PAGE separation on a 4–15% SDS–PAA gel and western blotting.

Immunoprecipitation at endogenous level of FOXA1, TET1, and TET2 was obtained by incubating rabbit anti-FOXA1 polyclonal, mouse anti-TET1 monoclonal, and rabbit anti-TET2 monoclonal antibodies, respectively, coupled with protein G Dynabeads (Invitrogen) with nuclear extract derived from MCF-7 cells for 2 h, with rotation at 4 °C. As negative controls, mouse or rabbit normal IgG coupled with protein G Dynabeads were used. Prior to incubation, we washed the beads once with 1 × PBS supplemented with 0.03 µg BSA and further blocked them with 0.03 µg BSA in 1 × PBS for 10 min with rotation. We then washed the beads twice with 400 µl lysis buffer (20 mM HEPES, 10% Glycerol, 0,05%NP-40, 1,5 mM MgCl_2_, 150 mM KAc, and 1 mM DTT supplemented with 5 × Complete protease inhibitor cocktail). Each wash was performed for 5 min with rotation at 4 °C. The bound proteins were eluted with a 20 μl 3 × SDS loading buffer at 95 °C for 10 min. After SDS–PAGE separation on a 4–15% SDS–PAA gel, the proteins were detected with western blot using a OdysseyCLX (LI COR).

## Supplementary Information

**Additional file 1:**  Differential TFBS enrichment for emCpGs vs. non-correlated CpGs.

**Additional file 2:** Differential TFBS enrichment for non-correlated CpGs vs. emCpGs.

**Additional file 3: Figure S1.** emQTL analysis identified TFs associated with DNA methylation patterns in TCGA cancer types.** Figure S2.** Assessment of the overlap between emCpGs predicted for the same emTF between cancer types. ** Figure S3.** Assessment of the overlap between emCpGs predicted for the same emTF between cancer types. ** Figure S4.** Functional genomic regions distribution of emCpGs and the complete set of CpGs considered for the emQTL analysis (n=376,997). ** Figure S5.** Distribution of Spearman rho values for emCpGs. ** Figure S6.** Distribution of Spearman rho values between methylation at emCpGs and ATAC signal. ** Figure S7.** GC content and DNA methylation profiles for emCpGs and non-correlated CpGs close to the TFBSs of the emTFs.** Figure S8.** Same as Fig. S7 considering BHLHE40, ETS1, and FLI1 in the BRCA cohort. ** Figure S9.** Same as Fig. S7 considering GATA3, RUNX3, and SPI1 in the BRCA cohort. ** Figure S10.** Same as Fig. S7 considering SOX2 in the HNSC cohort, and FLI1 and SPI1 in the KIRP cohort. ** Figure S11.** Same as Fig. S7 considering BHLHE40, CEBPB, and ETS1 in the LGG cohort. ** Figure S12.** Same as Fig. S7 considering PBX3, RUNX1, and RUNX3 in the LGG cohort. ** Figure S13.** Same as Fig. S7 considering SOX2 and SPI1 in the LGG cohort and FOXA2 in the LUAD cohort. ** Figure S14.** Same as Fig. S7 considering SOX2 and TP63 in the LUSC cohort, and CEBPB in the PRAD cohort. ** Figure S15.** Same as Fig. S7 considering RUNX1 and TP63 in the PRAD cohort, and SPI1 in the SKCM cohort.** Figure S16.** Same as Fig. S7 considering GATA3 in the STAD cohort, and PBX3 and RUNX1 in the THCA cohort. ** Figure S17.** Same as Fig. S7 considering SPI1 in the THCA cohort and FOXA2 in the UCEC cohort. ** Figure S18.** GO enrichment for gene targets of emCpGs. ** Figure S19.** Western blot validations of siFOXA1 and transient rescue. ** Figure S20.** Comparison between mTET2 and hTET2.

**Additional file 4: Table S1.** Number of samples (patients) corresponding to RNA-seq and 450 K methylation array data from TCGA.** Table S2.** Number of CpGs per TF that are found close to UnBind TFBSs.** Table S3.** identified emQTLs (TF-to-CpG associations).** Table S4.** Known pioneer TFs.** Table S5.** Flanking accessibility associated TFs.** Table S6.** Percentage of CpGs linked to target genes using STITCHIT and a distance-based approach.

## Data Availability

The code used to generate the emQTL analysis and the processing of the methylation array data is available at https://bitbucket.org/CBGR/tfme/src/master/. The raw and processed methylation data are available on GEO with the accession number GSE174008.
